# Supplementary material for: A Catalog of over 5,000 Metagenome-Assembled Microbial Genomes from the Caprinae Gut Microbiota
Source: Microbiol Spectr. 2022 Nov 2;10(6):e02211-22. doi: 10.1128/spectrum.02211-22 (PMC9769736; doi:10.1128/spectrum.02211-22)
Supplement: Supplemental file 3 — Fig. S1-S7. Download spectrum.02211-22-s0003.pdf, PDF file, 0.6 MB [file spectrum.02211-22-s0003.pdf]

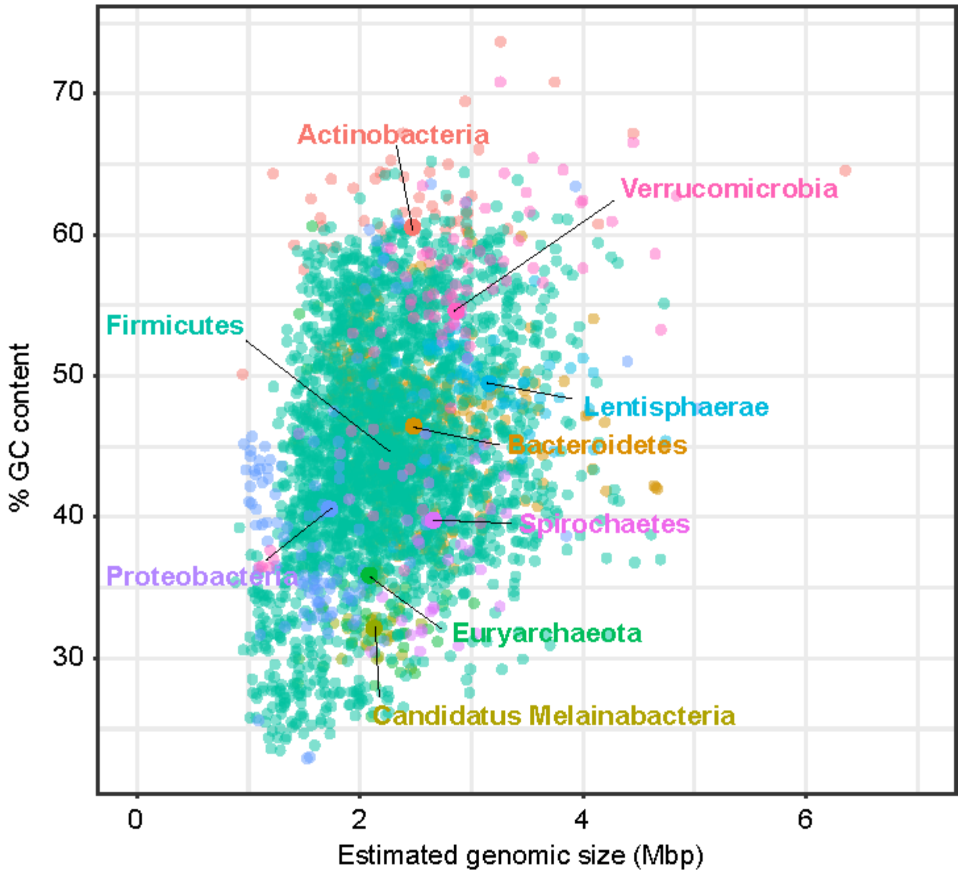

Figure S1 GC content and genome size of MAGs at phylum level.

Different colors represent different phyla, and the center point represents group mean value.

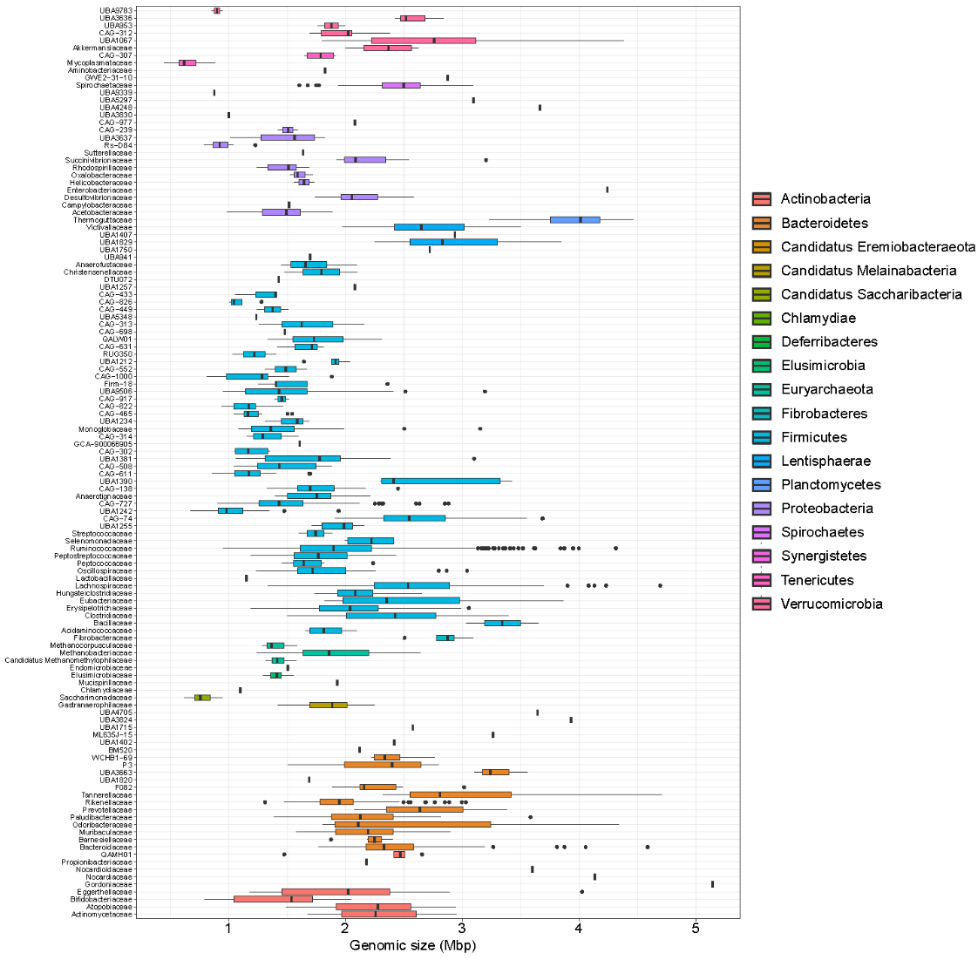

Figure S2 Genome size of MAGs at family level.

The boxes represent the interquartile range between the first and third quartiles and median; whiskers denote the minimum and maximum values within 1.5 times the range of the first and third quartiles, respectively; and bold dot represent outliers beyond the whiskers. The color fills represent different phyla levels, and the boxes with same color were different families.

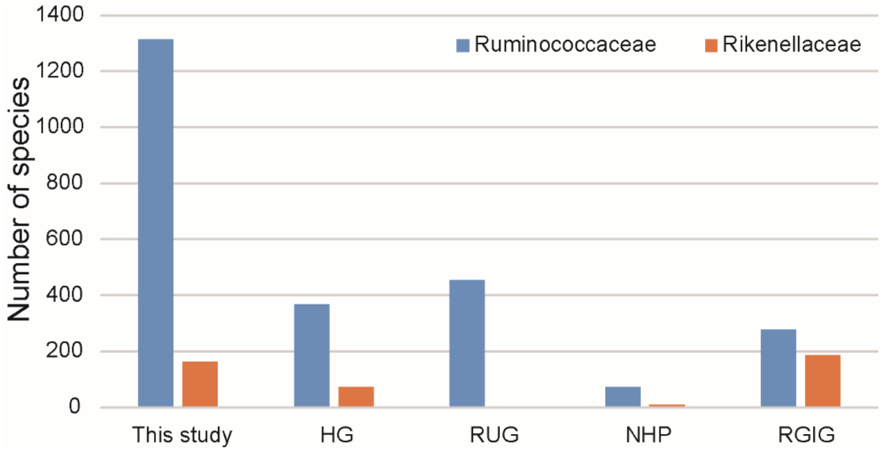

Figure S3 The number of species in Ruminococcaceae and Rikenellaceae between different data sets.

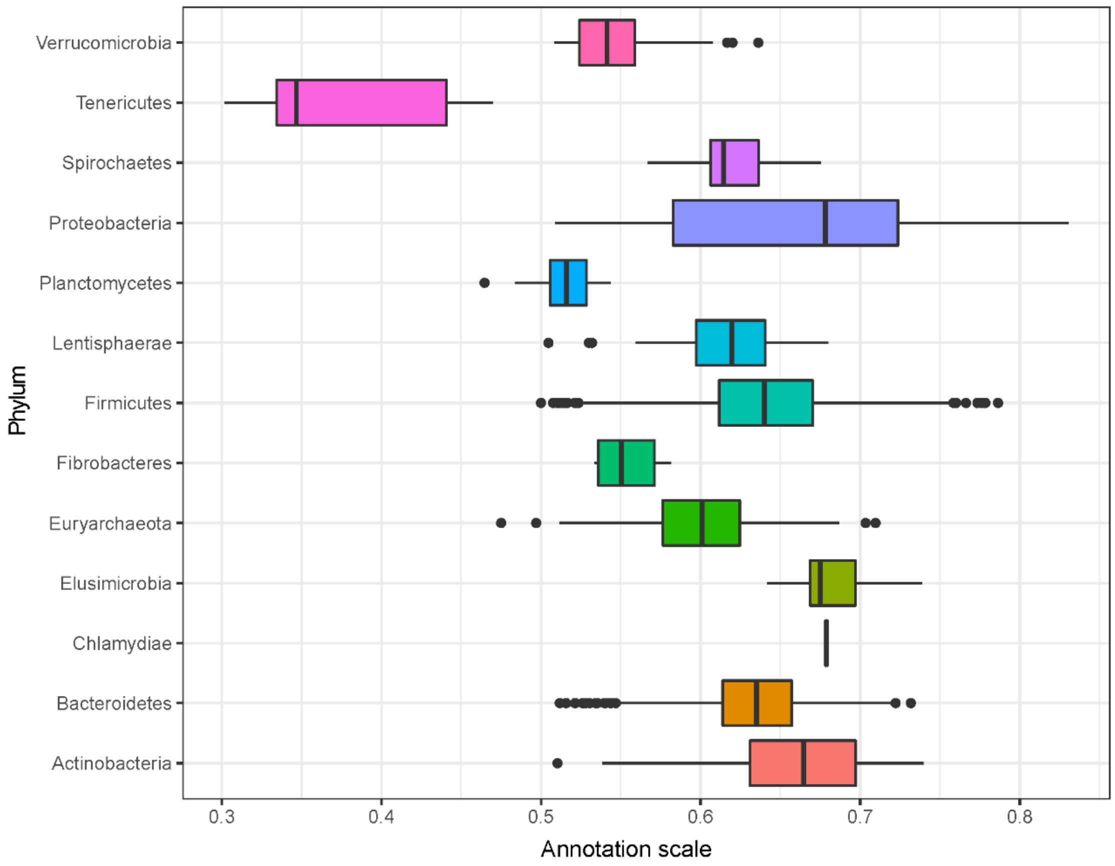

Figure S4 The proportion of genes classified to KEGG function was at the phylum level.

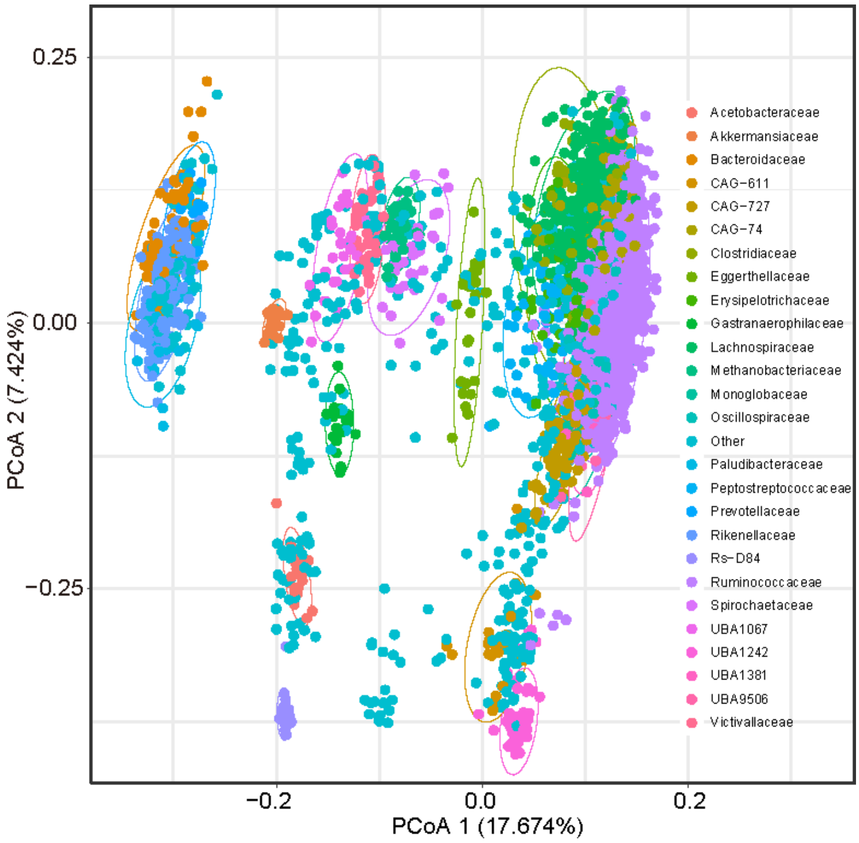

Figure S5 Principal coordinates analysis (PCoA) of Bray-Curtis distance on the KOs. Color padding indicates different families, and the ellipse represents the 95% confidence interval range.

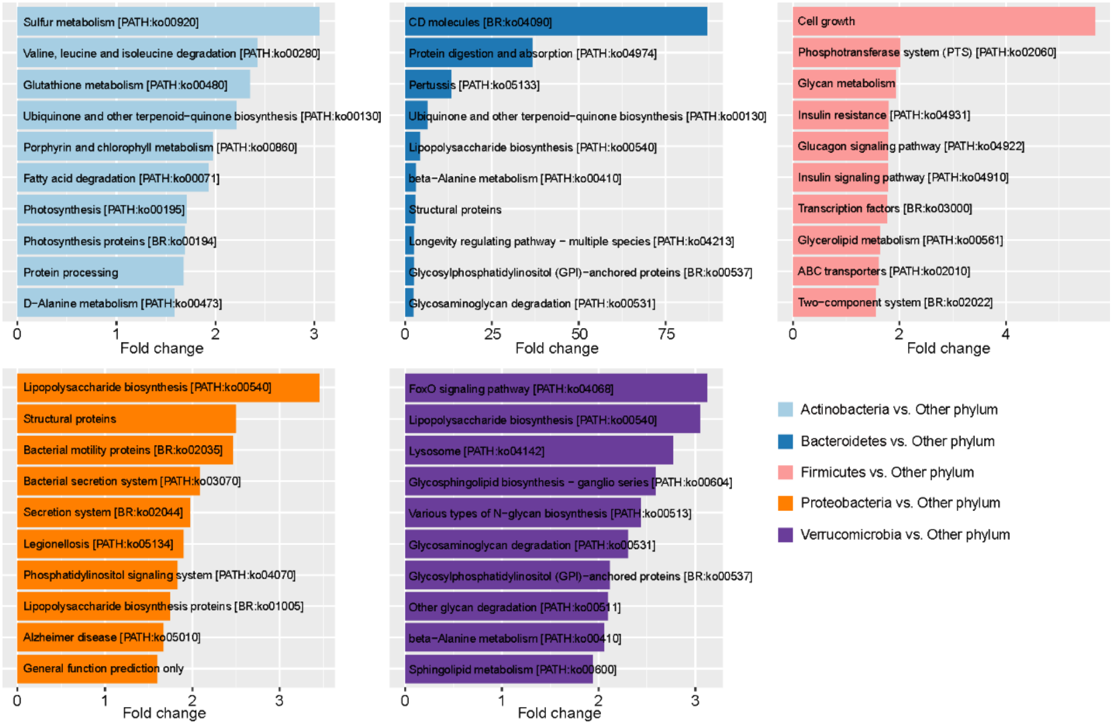

Figure S6 KEGG functional characteristics of five major phyla of gut microbial (vs. another

phyla)

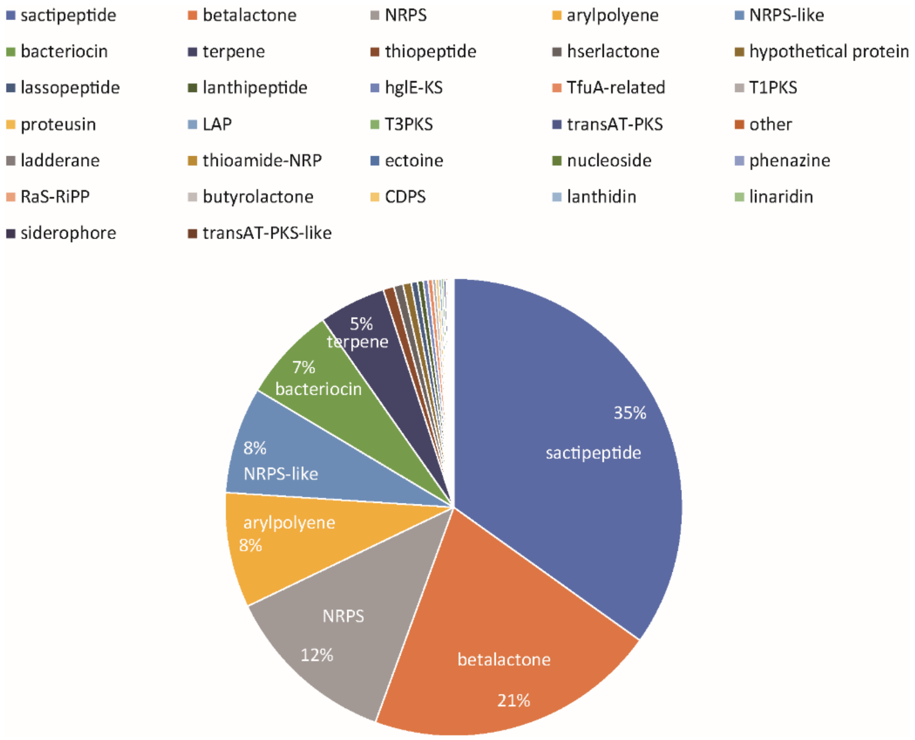

Figure S7 Classification and proportion of secondary metabolite biosynthesis gene cluster (BGCs) in gut species.
